# The Impact of the FIFA 11+ Injury Prevention Program on Injury Incidence in Football Athletes: A Systematic Review of Randomized Controlled Trials

**DOI:** 10.7759/cureus.100463

**Published:** 2025-12-31

**Authors:** Purvi Patel, Malkesh Shah

**Affiliations:** 1 Physiotherapy, Sumandeep Vidyapeeth, Vadodara, IND; 2 Orthopaedics, Sumandeep Vidyapeeth, Vadodara, IND

**Keywords:** fifa 11+, football (soccer), injury incidence, injury prevention, lower extremity injuries, neuromuscular warm-up, sports injuries

## Abstract

Football is associated with a high incidence of lower-extremity injuries, creating a substantial burden on athletes, teams, and healthcare systems. The Fédération Internationale de Football Association *(*FIFA) 11+ warm-up program was developed as a structured neuromuscular training routine to reduce injury risk by improving strength, balance, proprioception, and movement control. This systematic review synthesised controlled trials evaluating the effectiveness of the standard FIFA 11+ program among football players across youth, collegiate, and adult levels. Electronic databases were searched from 2008 to 2025 following Preferred Reporting Items for Systematic Reviews and Meta-Analyses (PRISMA) guidelines. Five controlled trials met the inclusion criteria. Due to heterogeneity in injury definitions, adherence reporting, and exposure-hour documentation, a narrative synthesis was performed. Across all studies, teams performing the FIFA 11+ demonstrated meaningful reductions in overall and lower-extremity injury incidence compared with those using traditional warm-ups, with preventive effects ranging from approximately 30% to 46%. The program’s effectiveness appeared to be mediated by improvements in neuromuscular control, trunk and hip stability, eccentric strength, and dynamic alignment during high-risk football movements such as cutting and landing. Adherence emerged as the primary determinant of success. These findings support the FIFA 11+ as an effective, low-cost, and accessible strategy for reducing football injuries when implemented consistently.

## Introduction and background

Football (soccer) is the most widely played sport globally, involving more than 270 million participants across youth, amateur, and elite competitive levels [1]. Although participation in football provides significant physical and psychological benefits, it is associated with a high incidence of musculoskeletal injuries, particularly of the lower extremities. Epidemiological studies report injury rates ranging from seven to 40 injuries per 1,000 hours of exposure, with most injuries occurring during high-speed actions such as acceleration, deceleration, cutting, and landing [2]. Youth and collegiate athletes may be especially vulnerable due to ongoing physical maturation, neuromuscular imbalance, increased training loads, and rapid changes in growth velocity [3]. These injuries result in time-loss from sport, reduced performance, and substantial economic burden for athletes, clubs, and healthcare systems, underscoring the importance of effective injury-prevention strategies.

In response to the growing need for structured and evidence-based warm-up interventions, the Fédération Internationale de Football Association (FIFA) Medical Assessment and Research Centre (F-MARC) developed the FIFA 11+ program [4]. This standardized warm-up routine aims to reduce injury risk by enhancing neuromuscular control, strength, balance, core stability, agility, and movement technique. The program comprises three components: (1) running and activation exercises, (2) strength, balance, and plyometric tasks emphasizing eccentric hamstring loading and dynamic knee stability, and (3) advanced sport-specific running drills focusing on safe direction changes and landing mechanics [5]. The program is designed to replace traditional warm-ups and requires minimal equipment, facilitating implementation across varying competitive and resource environments.

Since its introduction, the FIFA 11+ has been widely promoted and incorporated into training protocols by football clubs, national federations, and grassroots development programs. Implementation initiatives have focused on coach education, physiotherapist involvement, and adherence monitoring, given that the effectiveness of neuromuscular warm-ups is closely linked to consistent and correct execution [6]. Although numerous injury-prevention approaches exist in team sports, the FIFA 11+ is distinguished by its structured progression, global dissemination, and strong foundation in neuromuscular and biomechanical principles [7].

Despite its widespread adoption, important questions remain regarding real-world effectiveness. Injury profiles differ across age groups, competitive levels, and countries, raising concerns about the generalizability of existing evidence [5,7]. Furthermore, previous systematic reviews have often included heterogeneous populations, such as referees, children performing modified versions of the program, or athletes from other sports, which limits direct applicability to football-specific settings [8]. Additionally, controlled trials published in the past decade across diverse regions have expanded the evidence base but have not been synthesized in a focused, football-specific manner. Although several systematic reviews have examined the FIFA 11+ injury-prevention program, many have included mixed-sport populations, modified versions of the program, or non-controlled study designs. The present review is intentionally restricted to controlled trials conducted exclusively in football players using the standard FIFA 11+ program, thereby enhancing clinical specificity and applicability. In addition, this review emphasizes adherence and real-world implementation as key determinants of injury reduction.

To address these gaps, a systematic review restricted exclusively to controlled trials involving football players is warranted. Such a sport-specific synthesis enhances clinical relevance by isolating evidence derived directly from football contexts and standardized FIFA 11+ implementation. The purpose of this systematic review is therefore to evaluate controlled trials examining the effect of the FIFA 11+ warm-up program on injury incidence among football athletes across youth, collegiate, amateur, and adult competitive levels.

## Review

This systematic review followed the Preferred Reporting Items for Systematic Reviews and Meta-Analyses (PRISMA) 2020 guidelines [9]. All methodological processes, including the search strategy, eligibility criteria, and synthesis plan, were established prior to article screening to ensure transparency and methodological rigor. This systematic review was prospectively registered with the International Prospective Register of Systematic Reviews (PROSPERO) registration number: CRD42025126679.

Search strategy

Electronic databases, including PubMed, Scopus, Web of Science, PEDro, and Google Scholar, were systematically searched from January 2008 through March 2025, corresponding to the period following the development and dissemination of the FIFA 11+ program. The search strategy incorporated combinations of the keywords “FIFA 11+,” “FIFA 11 plus,” and “11+ injury prevention,” together with the terms “football,” “soccer,” “injury,” “injury prevention,” “trial,” “controlled,” and “randomized,” using Boolean operators as appropriate. These terms were adapted for each database to maximize sensitivity and comprehensiveness. In addition, reference lists of included studies and relevant reviews were manually screened to identify further eligible publications.

Eligibility criteria

Studies were considered eligible if they involved football or soccer athletes at the youth, collegiate, amateur, or adult competitive level; implemented the standard FIFA 11+ warm-up program as the primary intervention; included a comparator group performing a usual warm-up or alternative structured warm-up; and reported outcomes related to injury incidence, lower-extremity injury rates, or time-loss injuries. Only randomized controlled trials or controlled trials published in English between 2008 and 2025 were included. Studies were excluded if they investigated modified FIFA 11+ Kids or “11+ Referee” programs, involved mixed-sport samples, focused exclusively on biomechanical or laboratory outcomes without injury data, or lacked an appropriate control group. Review articles, editorials, and conference abstracts were also excluded.

Study selection

**Figure 1:**
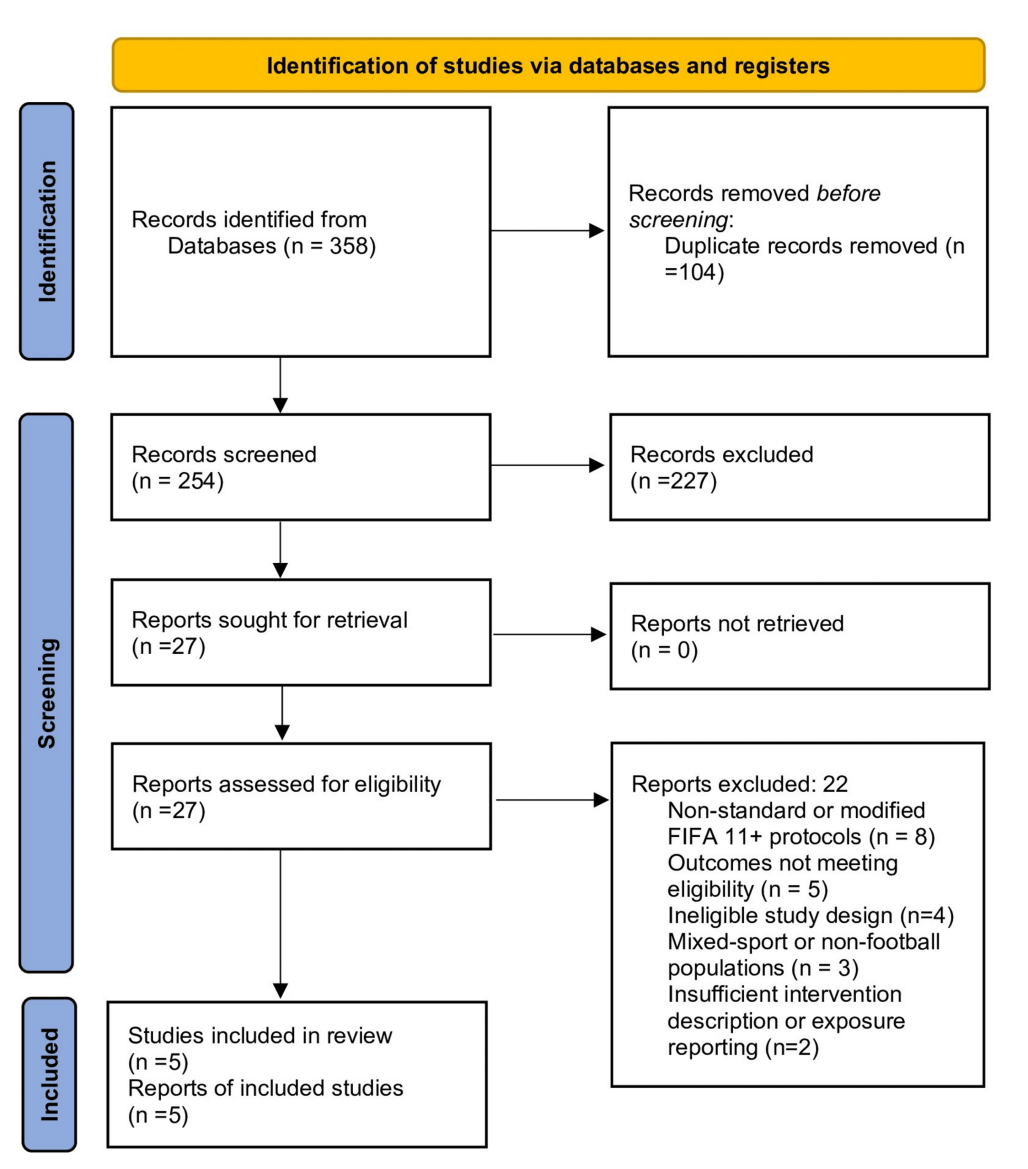
PRISMA 2020 flow diagram of study selection PRISMA: Preferred Reporting Items for Systematic Reviews and Meta-Analyses

The electronic database search identified 358 records. After removal of 104 duplicate citations, 254 titles and abstracts were screened for eligibility. Of these, 227 records were excluded for failing to meet the inclusion criteria. Twenty-seven full-text articles were assessed for eligibility, of which 22 studies were excluded due to the use of non-standard versions of the FIFA 11+ program, absence of injury-related outcomes, mixed-sport populations, or inappropriate study design. Ultimately, five controlled trials met all eligibility criteria and were included in the final synthesis (Figure 1).

Data extraction

Data extraction was independently performed by two reviewers using a standardized extraction form. Extracted information included study design, country of origin, participant characteristics, intervention duration and frequency, comparator warm-up protocols, injury definitions, exposure reporting methods, adherence rates, and primary injury outcomes. Disagreements between reviewers were resolved through discussion to achieve consensus and ensure accuracy.

Quality assessment

The methodological quality of the included studies was assessed using the Physiotherapy Evidence Database (PEDro) scale [10], as all included investigations employed randomized or cluster-randomized controlled trial designs. The assessment focused on randomization procedures, allocation concealment, blinding of outcome assessors, completeness of follow-up, and clarity of statistical reporting. Blinding of participants and coaches was not feasible due to the nature of the intervention. 

Data synthesis

Due to heterogeneity in injury definitions, exposure-hour reporting, adherence monitoring, and statistical outcome measures across studies, a meta-analysis was not appropriate. Therefore, a narrative synthesis approach was adopted, focusing on overall injury reduction, lower-extremity injury patterns, adherence-related effects, and implementation characteristics observed across the included trials.

Results

Study Selection

A total of five controlled trials met the eligibility criteria and were included in the synthesis (Table 1) [7,11-14]. The study selection process is summarized in the PRISMA flow diagram (Figure 1).

**Table 1:** Summary of published controlled trials (2008–2025) evaluating the FIFA 11+ injury prevention program in football athletes RCT: Randomized controlled trial, FIFA: Fédération Internationale de Football Association.

Author (Year)	Study role	Study design	Population	Intervention duration	Primary outcome	Key findings
Soligård et al. (2008) [11]	Primary efficacy trial	Cluster-RCT	Youth female football players	Full competitive season	Injury incidence	Significant reduction in overall and lower-extremity injuries in FIFA 11+ group
Steffen et al. (2013) [7]	Implementation / adherence study	Controlled trial	Youth female football players	Competitive season	Compliance, injury risk	Injury reduction strongly dependent on adherence; high-compliance teams benefited most
Owoeye et al. (2014) [12]	Primary efficacy trial	Cluster-RCT	Youth male football players	Competitive season	Injury incidence	41% reduction in overall injuries; fewer lower-limb injuries
Silvers-Granelli et al. (2015) [13]	Primary efficacy trial	RCT	Collegiate male football players	Competitive season	Injury incidence, time-loss injuries	46% reduction in total injuries; fewer time-loss injuries
Nuhu et al. (2021) [14]	Primary efficacy trial	Cluster-RCT	Adult competitive football players	Competitive season	Injury incidence and severity	Significant reductions in injury incidence and injury severity

Study Characteristics

Included studies investigated football players across youth, collegiate, and adult competitive levels. Participants comprised young female players, young male players, collegiate male athletes, and adult competitive footballers. All studies implemented the standard FIFA 11+ warm-up program and compared it with usual team warm-up routines. Study designs were predominantly cluster-randomized controlled trials, with interventions delivered across a full competitive season. Injury surveillance was conducted prospectively by trained medical staff or designated team personnel using standard football injury definitions. Methodological quality and adherence characteristics of the included studies are summarized in Table 2, with adherence classified according to session frequency, duration, or compliance thresholds as reported by the original study authors.

**Table 2:** Methodological quality and adherence characteristics of included studies PEDro: Physiotherapy Evidence Database, RCT: Randomized controlled trial.

Study	Study design	Quality assessment tool	Score	Risk of bias	Adherence / compliance reporting
Soligård et al. (2008) [11]	Cluster RCT	PEDro	7/10	Low	Reported; high compliance (≥2 sessions/week)
Steffen et al. (2013) [7]	Cluster RCT (implementation)	PEDro	6/10	Moderate	Reported; compliance-dependent outcomes
Owoeye et al. (2014) [12]	Cluster RCT	PEDro	7/10	Low	Reported; moderate–high adherence
Silvers-Granelli et al. (2015) [13]	Cluster RCT	PEDro	8/10	Low	Reported; high adherence throughout season
Nuhu et al. (2021) [14]	Cluster RCT	PEDro	6/10	Moderate	Reported; adherence monitored but variable

Narrative Synthesis

Across the included controlled trials, the FIFA 11+ warm-up program was consistently associated with reduced injury incidence compared with traditional warm-up routines. Four trials evaluated injury reduction as the primary outcome. In youth female football players, Soligård et al. (2008) demonstrated significant reductions in overall and lower-extremity injuries following implementation of the FIFA 11+ program [11].

Owoeye et al. (2014) reported a 41% reduction in overall injury incidence and substantial decreases in lower-limb injuries. In collegiate male football players among young male athletes [12], Silvers-Granelli et al. (2015) observed a 46% reduction in total injuries and fewer time-loss injuries in teams performing the FIFA 11+ compared with controls [13]. In adult competitive footballers, Nuhu et al. (2021) reported significant reductions in both injury incidence and injury severity [14]. In addition to these efficacy trials, Steffen et al. (2013) evaluated implementation strategies and adherence to the FIFA 11+ program in youth female football [7]. Although injury reduction was not the primary outcome, the study demonstrated that teams with high compliance achieved significantly greater injury reduction than those with poor adherence. These findings highlight adherence as a critical determinant of the program’s preventive effectiveness.

Discussion

Summary of Main Findings

This systematic review synthesized evidence from five controlled studies evaluating the FIFA 11+ injury-prevention program in football players, including four primary efficacy trials and one implementation-focused study. Across the primary trials, the FIFA 11+ consistently demonstrated clinically meaningful reductions in overall and lower-extremity injury incidence, with reductions typically ranging from 30% to 46% [7,11-14]. The consistency of these findings across youth, collegiate, and adult football populations provides strong support for the effectiveness of the program when implemented as part of routine training.

Effectiveness of the FIFA 11+ Program

Football injuries most commonly occur during non-contact situations involving rapid deceleration, cutting, pivoting, or landing. These actions place high demands on neuromuscular coordination, trunk stability, and lower-limb alignment, particularly under fatigue. Deficits in these domains are well-established contributors to injury risk in football athletes [15,16]. The FIFA 11+ program directly addresses these mechanisms through exercises that improve dynamic balance, eccentric hamstring strength, hip and trunk stability, and movement control during football-specific tasks [17]. 

Repeated exposure to controlled strength, balance, and plyometric exercises promotes favorable neuromuscular adaptations, improving joint positioning and intermuscular coordination. Such adaptations reduce exposure to high-risk biomechanical positions during match play, even under unpredictable, fatigued conditions. Neuromuscular warm-up programs combining balance, strength, plyometrics, and movement retraining have consistently been shown to reduce non-contact injury risk by improving lower-limb alignment and motor control during sport-specific activities [18,19]. The consistent injury reductions observed across all primary trials in this review suggest that these mechanisms translate effectively into real-world football environments.

Consistency Across Age Groups and Competitive Levels

An important finding of this review is the effectiveness of the FIFA 11+ across a wide range of football populations. Youth female players, youth male players, collegiate athletes, and adult competitive footballers all demonstrated significant reductions in injury incidence following implementation of the program [11-14]. This consistency suggests that the neuromuscular deficits targeted by the FIFA 11+ are common across developmental stages and levels of play.

The program’s structured progression allows it to remain appropriate despite differences in physical maturity, training intensity, and match demands. Core components such as balance, trunk control, eccentric strength, and movement quality are universally relevant to football performance and injury prevention. This broad applicability supports the use of the FIFA 11+ as a standardized injury-prevention strategy across entire football development pathways, from grassroots academies to senior competitive teams.

Role of Adherence and Implementation

While the primary efficacy trials establish that the FIFA 11+ is capable of reducing injuries, the implementation-focused study by Steffen et al. [7] provides important insight into real-world effectiveness. This study demonstrated that injury-reduction benefits were strongly dependent on adherence, with high-compliance teams achieving substantially greater reductions in injury risk than teams with poor compliance. These findings highlight that the FIFA 11+ is a dose-dependent intervention; consistent exposure, correct technique, and appropriate weekly frequency are essential for neuromuscular adaptations to occur.

Evidence from implementation research further suggests that coach education, leadership support, and integration of injury-prevention exercises into routine training structures are critical factors influencing long-term adherence [17]. Without these supportive strategies, even well-designed programs may fail to achieve their intended preventive effects. These findings emphasize that implementation fidelity is as important as program content in achieving injury-prevention benefits.

Clinical and Practical Implications

From a physiotherapy and sports medicine perspective, the FIFA 11+ represents a highly practical injury-prevention strategy. It is low-cost, requires minimal equipment, and can be completed within 15-20 minutes as part of a standard warm-up. In addition to injury reduction, neuromuscular warm-up programs similar to the FIFA 11+ have been associated with improvements in performance-related attributes such as sprint speed, jumping ability, and agility, indicating that injury prevention and performance enhancement may coexist [18]. For clinicians, the exercises within the FIFA 11+ also allow observation of movement quality, balance deficits, and asymmetries, which may help identify players at increased injury risk. Because many of the exercises overlap with early rehabilitation and return-to-play principles, the program offers continuity between injury prevention and rehabilitation.

Interpretation Within the Broader Injury-Prevention Literature

The findings of this review are consistent with broader evidence demonstrating that neuromuscular training programs reduce lower-extremity injury risk across various sporting populations. Meta-analyses have shown that structured neuromuscular interventions can substantially reduce non-contact injury risk, particularly when programs are well designed and consistently implemented [19-21]. The FIFA 11+ aligns closely with these evidence-based principles, which likely explains its robust preventive effects across different football contexts.

Limitations

The included studies varied in injury definitions, exposure-hour reporting, and adherence measurement, which limited direct comparability and precluded meta-analysis. In addition, most trials followed players for a single competitive season, restricting conclusions regarding long-term injury prevention.

Future directions

Future research should prioritize multi-season evaluations, standardized injury surveillance methodologies, and implementation-focused strategies aimed at improving long-term adherence and delivery fidelity across football programs.

## Conclusions

This systematic review indicates that the FIFA 11+ warm-up program is an effective and practical strategy for reducing injury incidence in football players across youth, collegiate, and adult competitive levels. Across controlled trials, consistent reductions in overall and lower-extremity injury incidence were reported, typically ranging from 30% to 46%, likely mediated by improvements in neuromuscular control, trunk and hip stability, eccentric strength, dynamic balance, and movement mechanics during high-risk football activities. Adherence emerged as the key determinant of effectiveness, with regular and correctly performed sessions required to achieve meaningful injury reduction. Given its low cost, minimal equipment requirements, and ease of integration into routine training, the FIFA 11+ can be feasibly implemented across football settings and is supported as a standard component of football warm-up practice to enhance player safety and reduce preventable injuries.
